# Beyond the hype: Antibody-Drug Conjugates are advancing faster than our clinical strategy

**DOI:** 10.1371/journal.pmed.1004930

**Published:** 2026-01-30

**Authors:** Giulia Notini, Giampaolo Bianchini, José Manuel Pérez-García, Javier Cortés

**Affiliations:** 1 Department of Medical Oncology, IRCCS San Raffaele Hospital, Milan, Italy; 2 School of Medicine and Surgery, Vita-Salute San Raffaele University, Milan, Italy; 3 International Breast Cancer Center (IBCC), Pangaea Oncology, Quiron Group, Barcelona, Spain; 4 Medica Scientia Innovation Research (MEDSIR), Barcelona, Spain; 5 IOB Madrid, Institute of Oncology, Hospital Beata María Ana, Madrid, Spain; 6 Oncology Department, Hospital Universitario Torrejón, Ribera Salud Group, Madrid, Spain; 7 Department of Medicine, Faculty of Biomedical and Health Sciences, Universidad Europea de Madrid, Madrid, Spain

## Abstract

In this Perspective, Giulia Notini and colleagues discuss why the rapid expansion of antibody-drug conjugates, while redefining treatment options in advanced breast cancer, has also resulted in several unresolved challenges, including the need for rational sequencing strategies, appropriate and ethical trial design, drug tolerability, and the limitation of mono-national development programs.

## Teaser

Antibody-drug conjugates are redefining treatment options in advanced breast cancer, demonstrating efficacy across all breast cancer subtypes. However, their rapid clinical expansion has resulted in several unresolved challenges, including the need for rational sequencing strategies, appropriate and ethical trial design, drug tolerability, and the limitation of mono-national development programs.

## Introduction

Systemic treatment for advanced breast cancer has traditionally relied on chemotherapy, immunotherapy, endocrine therapy, and targeted agents, depending on tumor subtype. Despite substantial progress, many patients derive only transient benefit from systemic therapies, with disease control frequently limited by primary or acquired resistance and by cumulative treatment-related toxicity. These challenges are particularly evident in biologically aggressive subtypes such as triple-negative breast cancer (TNBC).

Antibody-drug conjugates (ADCs), which link a tumor-targeting antibody to a potent cytotoxic payload, were developed to enhance drug delivery while reducing off-target exposure [1]. In less than a decade, ADCs have moved from late-line salvage options to central components of breast cancer management. First-line pivotal trials have shown exceptional activity across multiple subtypes: Trastuzumab deruxtecan (T-DXd), a HER2-directed ADC, has extended progression-free survival beyond 40 months in advanced HER2-positive breast cancer [2], while sacituzumab govitecan (SG), a trophoblast cell-surface antigen 2 (Trop-2)-targeted ADC, has demonstrated superiority over conventional chemotherapy in advanced TNBC, with consistent efficacy irrespective of PD-L1 status (a biomarker commonly used for patient stratification and treatment selection in this setting) [3,4]. These findings have prompted accelerated evaluation of ADCs in the (neo)adjuvant setting. Trials such as DESTINY-Breast05 (NCT04622319) [5] and DESTINY-Breast11 (NCT05113251) [6] in HER2-positive early-stage breast cancer (both of which have already yielded positive efficacy readouts), along with multiple Trop-2-directed ADC programs in early-stage TNBC, seek to improve outcomes in clinical scenarios where conventional chemotherapy has reached a therapeutic plateau.

However, beneath this impressive momentum, the growing number of ADCs with heterogeneous targets and payloads advancing through clinical development risks saturating the therapeutic armamentarium across tumor types. Consequently, the field requires more innovative and strategically optimized trial designs, alongside a clearer delineation of resistance mechanisms and predictive biomarkers, including those informing toxicity risk, to enable the rational and efficient clinical deployment of ADCs.

Within this rapidly shifting treatment paradigm, several fundamental considerations arise: How to optimize therapeutic sequencing in the context of increasingly effective front-line regimens; how to design clinically appropriate and ethically robust trials; how to balance efficacy with patient-experienced toxicity; and how to ensure global applicability when many emerging ADCs are developed within mono-national research programs whose populations may not reflect global heterogeneity. Addressing these issues is essential to integrate ADCs into a sustainable and evidence-based therapeutic framework.

## Sequencing without strategy: A missed opportunity for optimization

Until now, ADC development has largely adhered to a linear “replacement model,” in which each new agent is evaluated against chemotherapy within a single treatment line. However, this paradigm will no longer be acceptable as ADCs become established standards of care, rendering chemotherapy an increasingly obsolete comparator.

Cross-resistance is likely when ADCs share the same target, but it may also arise when agents employ similar payloads. Preliminary clinical evidence, primarily from SG and T-DXd, suggests reduced activity when mechanistically related ADCs are administered in sequence [7,8]; however, these signals are derived from observational cohorts, with no prospective studies yet providing definitive validation. Preclinical data also remain limited, and studies of resistance mechanisms in patients treated with ADCs, particularly those involving paired biopsies, are likewise insufficient.

Distinguishing whether resistance is driven by target-related mechanisms or by payload-specific biology is therefore essential for designing rational sequencing strategies. Despite the absence of definitive clinical or preclinical data, an emerging hypothesis that requires rigorous validation is that early-onset resistance to an ADC may be predominantly mediated by target-related mechanisms, such as reduced antigen expression or impaired internalization, whereas late-emerging resistance may more closely reflect payload-specific processes [9].

It is critical to avoid trial designs that implement ADC sequencing without mechanistic justification or comprehensive assessment of cross-resistance biology. The field should move away from “one-size-fits-all” approaches that disregard target and payload heterogeneity, and instead toward evidence-based frameworks that tailor sequencing to the underlying biology of resistance and drug mechanism, incorporating multi-omic biomarker strategies, including transcriptomic, proteomic, and liquid biopsy platforms.

## Designing the right trial, the right way

The selection of primary endpoints and control arms is a key determinant of breast cancer clinical trial design. Although overall survival (OS) has traditionally been regarded as the most robust endpoint in advanced disease, its interpretability is increasingly challenged by ethically appropriate cross-over, regional disparities in access to effective post-progression therapies, and selection of control arms that do not fully reflect the most effective available standards of care. As a result, OS may either dilute true survival differences or exaggerate apparent benefits, as survival outcomes may be driven as much by trial design and comparator choice as by true therapeutic efficacy, complicating interpretation, cross-trial comparisons, and the global generalizability of trial results.

## The illusion of a “better tolerated” therapy

Although ADCs are often promoted as more tolerable than conventional chemotherapy, they exhibit a broad spectrum of on-target and off-target toxicities, and their target selectivity does not eliminate the risk of clinically meaningful harm. Adverse events range from life-threatening complications, such as T-DXd-associated interstitial lung disease, to highly burdensome effects, including ocular surface disorders, asthenia, nausea, diarrhea, and mucositis, each of which can markedly compromise functional status and health-related quality of life [10]. A comprehensive understanding of these toxicity profiles, together with proactive mitigation and monitoring strategies, is therefore essential for the safe and effective integration of ADCs into routine practice.

Nevertheless, toxicity alone does not fully capture the patient experience, and the route of administration—together with treatment burden, including the frequency and duration of intravenous infusions—have emerged as additional determinants of both tolerability and clinical therapeutic value. In this context, the development of subcutaneous delivery platforms for ADCs represents a promising strategy to enhance convenience and potentially broaden access.

Overall, patient-reported outcomes (PRO) data from pivotal ADC trials indicate that, although global health-related quality-of-life scores are often preserved during treatment, domain-level analyses consistently reveal clinically meaningful increases in symptom-specific burdens [11]. This divergence underscores a critical gap between aggregate quality-of-life metrics and the symptom experience captured by more detailed PRO subscales.

In an era in which multiple mechanistically similar ADCs are advancing through clinical development, it is increasingly important to evaluate whether health-related quality of life could, in selected contexts, serve as a primary endpoint in place of traditional efficacy metrics. This paradigm may be particularly relevant for ADCs that demonstrate comparable antitumor activity yet offer a more favorable safety and tolerability profile.

## Clinical trial geography: The rise of mono-national pipelines

Most ADCs that reshaped global practice, such as T-DXd and SG, were evaluated in multinational cohorts, allowing broader generalizability of their efficacy and safety profiles. However, the oncology drug-development landscape is undergoing a profound regulatory shift, driven in large part by the rapid expansion of clinical innovation within China [12]. An increasing number of novel ADCs are progressing through the Chinese regulatory ecosystem, while others are being acquired or licensed by Western biopharmaceutical companies for further global development.

While the results from studies conducted exclusively in China are scientifically compelling, their applicability beyond East Asia remains uncertain. Beyond heterogeneity in access to standard-of-care therapies, pharmacogenomic differences across populations, including UGT1A1 and CYP polymorphisms relevant to camptothecin-based payloads, may influence drug metabolism and, consequently, both efficacy and toxicity [13].

As predominantly China-based mono-national pipelines continue to expand, the global clinical community must determine what level of evidence is sufficient for extrapolation to other regions. Whether regulatory approval outside the country of origin should require multinational validation or, alternatively, robust bridging datasets capable of demonstrating that efficacy and safety reliably translate across diverse populations, is a critical question that now demands urgent and transparent debate.

## Conclusions

ADCs have substantially expanded therapeutic options in advanced breast cancer, yet major challenges remain regarding optimal sequencing, tolerability, and global generalizability. Progress in drug development must be matched by equal rigor and strategy. The development of next-generation ADCs, with novel targets and payloads, together with the incorporation of multi-omic biomarker approaches, patient-centered outcome measures, and diverse trial populations into innovative trial designs that move beyond conventional paradigms, will be essential to translating ADC revolution into durable and globally applicable clinical benefit.

## Abbreviations

ADC: antibody-drug conjugate
OS: overall survival
PRO: patient-reported outcomes
SG: sacituzumab govitecan
T-DXd: Trastuzumab deruxtecan
TNBC: triple-negative breast cancer
Trop-2: trophoblast cell-surface antigen 2
